# The accuracy of reconstruction of orbital wall fractures using prebent mesh versus patient specific implants: a randomized clinical trial

**DOI:** 10.1186/s12903-025-07082-z

**Published:** 2025-11-01

**Authors:** Mona S. Sheta, Wesam H. Elsaadany

**Affiliations:** Oral and Maxillofacial Surgery Department, Faculty of Dentistry, Tanta University, El-Giesh St., Tanta, Gharbia 31527 Egypt

**Keywords:** Orbital fractures/Surgery, Three-dimensional printing, Surgical mesh

## Abstract

**Background:**

Repairing fractures of the orbital wall and floor remains a complex challenge due to the intricate anatomy of the orbit and the limited visibility during surgery. With the advancement of digital technologies, tools such as DICOM (Digital Imaging and Communications in Medicine) now allow the creation of virtual mirrored models of the unaffected orbit, which can greatly assist in surgical planning and reconstruction. This study aimed to evaluate the accuracy of orbital wall reconstruction using patient-specific implants (PSIs) versus prebent titanium mesh.

**Materials and methods:**

A single-blind randomized controlled clinical trial was carried out on 28 patients with unilateral orbital fractures, who were randomly divided into two equal groups. Group I underwent orbital wall reconstruction using prebent titanium mesh, while Group II received titanium PSIs. Clinical and radiographic evaluations were carried out at 1 week, 1 month, and 3 months postoperatively to evaluate enophthalmos, ocular motility, diplopia, and the precision of orbital volume restoration.

**Results:**

The mean postoperative orbital volume was 27.48 ± 1.324 cm³ in Group I and 25.62 ± 1.492 cm³ in Group II. The difference in orbital volume between the reconstructed and intact orbit was significantly smaller in the PSI group (0.6543 ± 0.2767 cm³) compared to the pre-bent mesh group (1.666 ± 0.3884 cm³; *p* < 0.05).

**Conclusion:**

Patient-specific titanium implants significantly improved the accuracy of orbital volume restoration, particularly in cases involving large defects. Compared to pre-bent titanium mesh, PSIs reduced the need for revision surgery and offered superior volumetric outcomes. In contrast, prebent titanium mesh still provided satisfactory results for isolated single wall fractures and is likely to be cost-effective.

**Trial registration:**

This trial was retrospectively registered at ClinicalTrials.gov on March 05, 2024, under the registration number NCT06294535.

**Supplementary Information:**

The online version contains supplementary material available at 10.1186/s12903-025-07082-z.

## Introduction

Post-traumatic orbital defects are a common injury and are mainly caused by motor vehicle accidents and assaults [1–3]. The most frequent fractures are “blow-out fractures”, which fracture the orbital floor and/or wall while sparing the orbital rim [4]. Management of orbital wall fractures remains a significant challenge in maxillofacial surgery due to their high incidence and complexity, which correlates with cosmetic and functional complications [5–7]. Traditional orbital reconstruction procedures often rely on standard titanium plates, polymeric implants, or meshes [5, 8]. These conventional implants typically require intraoperative manual bending to adapt to the patient’s anatomy, which causes a significant challenge in accurately positioning the implant within the orbit, particularly in cases where the orbital shelf and/or intra-orbital buttress is absent due to trauma [7, 9]. In such circumstances, the lack of structural support can lead to implant instability or malposition. Standard orbital meshes have also shown limitations, especially in cases requiring cantilevered reconstruction [10]. The reported surgical revision rate using traditional orbital plates was between 17% and 87.5% in the literature [11, 12]. Successful orbital reconstruction essentially depends on restoring the native orbital volume and shape, which requires precise adaptation of the implant to the specific architecture of the injured structures [13]. Recent advances in computer-aided design and computer-aided manufacturing (CAD/CAM) technology have drawn the accuracy of facial bone reconstruction and have drawn the attention of surgeons interested in orbital reconstruction [5, 7, 14]. Several studies have documented the effective use of customized patient-specific implants (PSIs) in both facial and orbital reconstructions owing to their superior anatomical fit [14–16].

The primary aim of this study was to evaluate the accuracy of titanium PSIs in orbital reconstruction compared with prebent implants. This was done by comparing the mean orbital volume differences between the reconstructed and the intact orbits in each group. The null hypothesis (H₀) stated that there would be no statistically significant difference between the two groups regarding orbital volume reconstruction accuracy.

## Materials and methods

This single-blind randomized controlled trial included 28 patients diagnosed with unilateral orbital wall fractures. Study was conducted in accordance with CONSORT reporting guidelines [17] and extended over one year from December 2023 to December 2024. All surgical procedures were performed in the Department of Oral and Maxillofacial Surgery, Faculty of Dentistry, Tanta University. Patients were evaluated clinically and radiographically using computed tomography (CT) scans.

The study protocol was approved by the Research Ethics Committee of Tanta University Faculty of Dentistry (R-OS-11–23-3078). The study complied with the ethical principles outlined in the 1964 Declaration of Helsinki and its subsequent amendments. All patients were fully informed about the purpose and nature of the study, and written informed consent was obtained from each participant before treatment. The clinical trial was retrospectively registered at ClinicalTrials.gov (Number: NCT06294535).

### Eligibility criteria

**Fig. 1:**
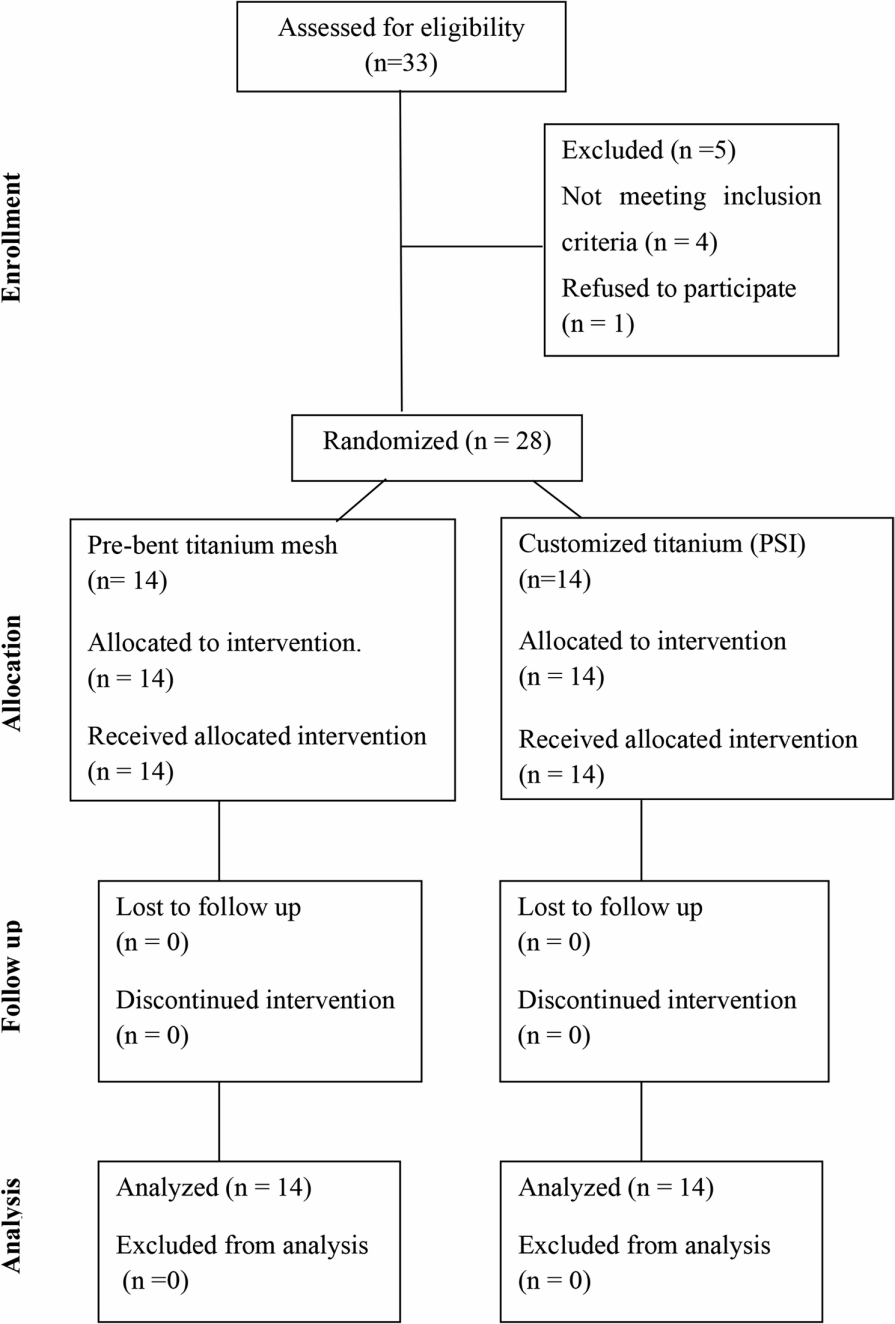
CONSORT flow chart

Patients were selected based on predefined inclusion and exclusion criteria. The inclusion criteria included unilateral orbital wall fractures associated with at least one of the following: diplopia within 30°, enophthalmos greater than 2 mm, or radiological evidence of extraocular muscle entrapment. The exclusion criteria included patients with bilateral orbital wall fractures, unassessable vision or diplopia, neurological disorders, or globe damage that prevented surgical reconstruction (Fig. 1).

### Sample size calculation, randomization, and blinding

G*Power Version 3.1.9.4 was used to calculate the sample size. The primary outcome of the power study was the difference in orbital volume between the reconstructed and unaffected orbit. According to the results of Sigron et al. [18], the effect size was determined to be 1.213. The study design included an alpha level of 5%, a beta level of 20%, and 80% power. Each group had an estimated sample size of 12 patients. To account for a 20% dropout rate, the sample size was increased to 14 patients in each group (for a total sample size of 28).

Twenty-eight patients were randomly assigned to two groups using sealed envelopes at a 1:1 allocation ratio (14 patients per group). In Group I: prebent titanium mesh was used to reconstruct the orbital wall. Group II: A customized titanium patient-specific implant (PSI) was applied to reconstruct the orbital wall. Blinding of operators and patients was not feasible. This was a single-blind study, in which blinding was limited to the investigator responsible for data analysis.

### Preoperative evaluation

Patients in both groups were examined preoperatively for visual acuity, ocular appearance, globe position, and ocular motility. Visual acuity was assessed using a standard distance eye chart. The external appearance of the eye, including the presence of hypoglobus was diagnosed by drawing an imaginary horizontal line through the patient’s inter-pupillary axis; a vertical displacement ≥ 2 mm from this line was considered clinically significant [19]. Enophthalmos was measured using a Hertel exophthalmometer [20]. Ocular motility limitation and diplopia were assessed by the “follow my finger” test, in which patients were instructed to follow the examiner’s finger in eight directions of gaze [21].

All patients underwent computed tomography (CT) scans preoperatively to assess the size and location of the defect as well as to assess the extent of any muscle or soft tissue entrapment. DICOM (digital imaging and communications in medicine) files from the CT images were imported into the Mimics^®^ medical 20.0 software (Materialise, Leuven, Belgium). Image segmentation was performed to highlight regions of interest and 3D reconstruction of the orbit, mirroring the unaffected orbit to generate a 3D reconstruction of the affected orbit using software 3 Matic 13 (Materialise, Leuven, Belgium). A 3D printer (Upbox, Tiertime, Korea) was used to print the patient specific model using the technology of fused deposition modelling.

According to preoperative CT data (DICOM files without compression), biomedical engineers established the design of the PSI in close collaboration with surgeons. The design was converted into an STL (stereolithography) file, which was sent to the engineer for milling. A CNC (computer numerical control) programming software was then used for manufacturing with a CAD/CAM milling machine (Millstar Jiuh-Yeh LMV 40 2 S) to fabricate titanium PSI.

### Surgical procedure

**Fig. 2:**
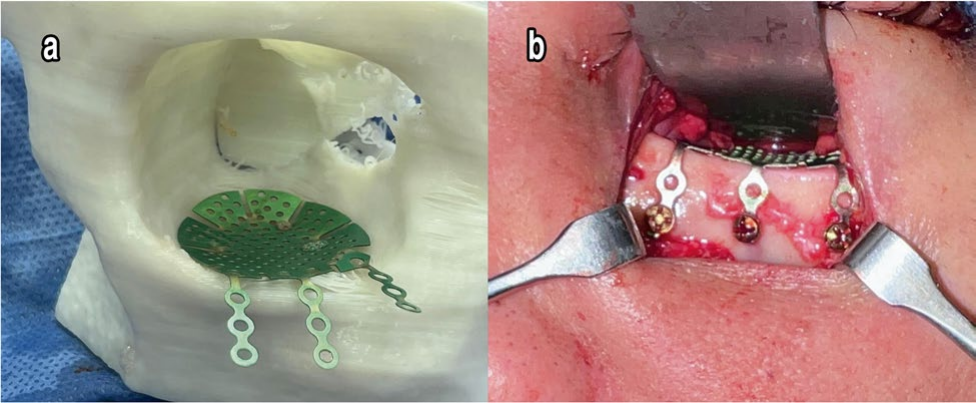
**a** Pre-bent titanium mesh was adapted on a 3D patient-specific orbital model, **b** Pre-bent titanium mesh was inserted into the orbit through an infraorbital incision (Group I)

**Fig. 3:**
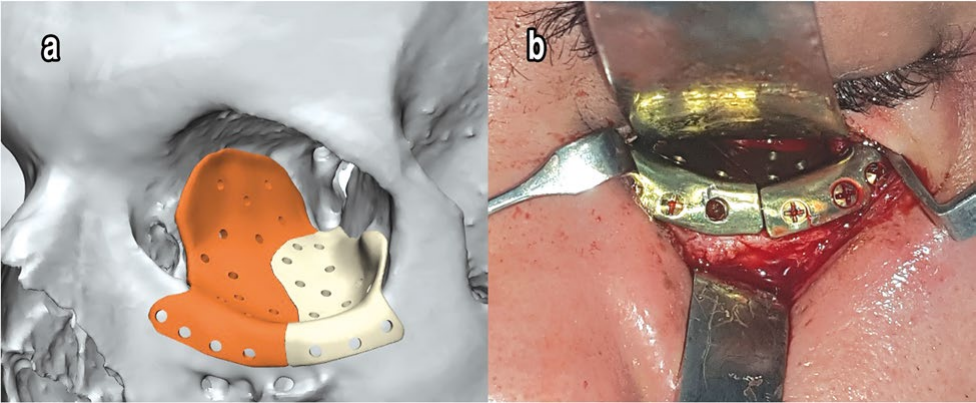
**a** Virtual planning of titanium PSI to reconstruct the orbital wall, **b** Titanium PSI after adapting within the orbit through infraorbital incision (Group II)

Two experienced oral and maxillofacial surgeons at the Department of Oral and Maxillofacial Surgery performed all surgeries. Under general anesthesia, a transcutaneous infraorbital incision was made to expose the fractured orbital floor and/or walls. After elevation of herniated orbital tissue, reconstruction was carried out according to the randomization result, the prebent titanium mesh (group I) or customized titanium patient-specific implant (PSI) (group II) was used to reconstruct the orbital walls and fixed to the inferior orbital rim using titanium miniscrews. Before closing the wound with cutaneous sutures, a forced duction test was performed to guarantee unrestricted passive movements of the eye (Figs. 2 and 3).

### Clinical assessment

The patients in both groups were evaluated clinically at one week, one month, and three months postoperatively for visual acuity, external eye appearance (including vertical hypoglobus and enophthalmos), and ocular motility. Limitations of ocular motility and diplopia were compared with preoperative baseline measurements to assess the postoperative clinical improvement.

### Radiographic assessment

Computed tomography (CT) scans were performed at 1 week and 3 months postoperatively to evaluate the accuracy of orbital reconstruction. Using Mimics^®^ Medical 20.0 software (Materialise, Leuven, Belgium), the postoperative reconstructed orbit was digitally superimposed onto the virtual mirrored model of the intact orbit. The orbital volume difference between the reconstructed and intact sides was calculated for each patient in both groups to estimate the precision of orbital reconstruction.

### Statistical analysis

Data was gathered, tabulated, and statistically analyzed with Statistical Package for Social Sciences (SPSS) version 26. For numerical data, Mann-Whitney test for inter-group comparison was used and Friedman test for intra-group comparison, using Dunn’s test for pairwise comparison when the p-value was equal or less than 0.05. Fisher’s exact test was used for nominal data for inter-group comparison.

## Results

### Demographic data

This study included 28 patients with unilateral orbital floor and/or wall fractures requiring surgical reconstruction, confirmed by clinical and radiographic evaluation. Patients were randomly allocated into two equal groups. Group I (*n* = 14) included 11 males and 3 females, aged between 19 and 57 years, who were treated using pre-bent titanium mesh. Group II (*n* = 14) included 12 males and 2 females, aged between 23 and 53 years, who underwent reconstruction using customized titanium PSIs. The mean surgical interval (time between trauma and surgical intervention) was 9.43 ± 1.79 days for Group I and 9.5 ± 1.66 days for Group II (Table 1). The most common mechanism of injury was road traffic accidents, accounting for 57.14% (*n* = 16) of cases, followed by assault in 25% (*n* = 7), and falls in 17.86% (*n* = 5).

**Table 1:** Demographic data

	Group I	Group II	*P*. value
Age			1ns"
M±SD	38.36±11.89	38.21±9.577	
(Min-Max)	(19-57)	(23-53)	
Sex, *n* (%)			0.2640ns*
Males *n* (%)	11(79%)	12(86 %)	
Females *n* (%)	3(21%)	2(14%)	
Surgical interval			0.9628ns"
M±SD	9.43±1.79	9.5±1.66	
(Min-Max)	(5-12)	(7-12)	

*Mann-Whitney test, "Fisher's exact test, *ns* not significant

### Clinical results

**Fig. 4:**
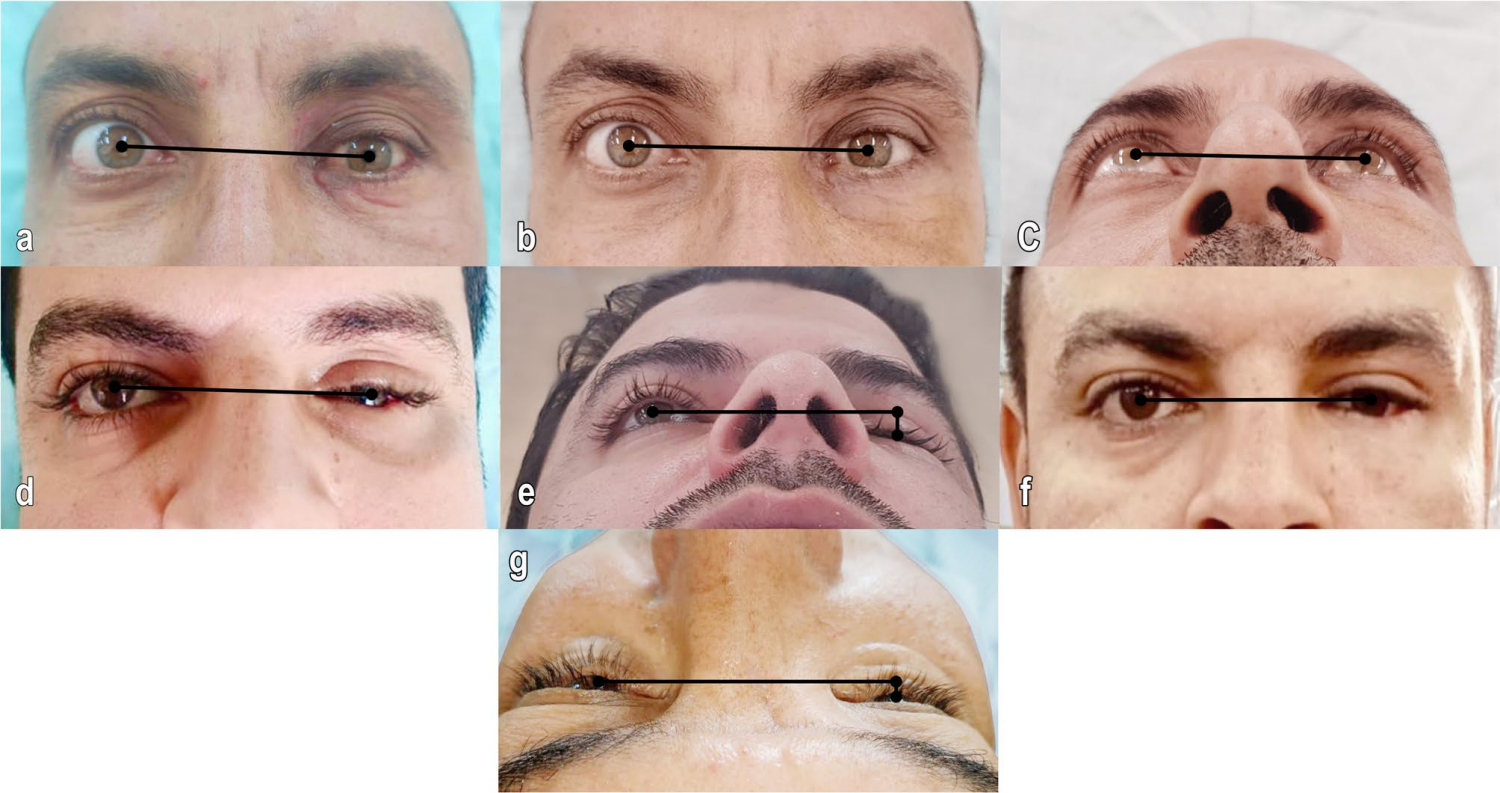
Group I, **a**) Preoperative enophthalmos and hypoglobus. **b **& **c** Postoperative clinical image showing improvement of hypoglobus and enophthalmos. Group II, **d** & **e** Preoperative photograph showing hypoglobus and significant enophthalmos of the left orbit. **f** Significant improvement of globe protrusion and vertical level of orbit three months after reconstruction. **g** Postoperative photograph showing residual enophthalmos

Throughout the postoperative follow-up period, no patients in either group experienced a decrease in visual acuity or loss of visual fields. Preoperatively, all patients in both groups presented with enophthalmos and hypoglobus (100%). Three months after surgery, only three patients (21%) in Group I had persistent enophthalmos, and two patients (14%) had persistent hypoglobus. In contrast, only one patient (7%) in Group II exhibited residual enophthalmos, while hypoglobus had completely resolved in all patients. (Fig. 4) and (Tables 2 and 3).

**Table 2:** The percentage of enophthalmos in both groups

Any enophthalmos *n* (%)	Preoperative Group I	Preoperative Group II	*P*. value	Postoperative Group I	Postoperative Group II	*P*. value
No *n* (%)	None (0%)	None (0%)	Not applicable	11 (79%)	13(93%)	0.0072***”
yes *n* (%)	14(100%)	14(100%)		3(21%)	1(7%)	

"Fisher's exact test, p***<0.001

**Table 3:** The percentage of hypoglobus in both groups

Any enophthalmos, *n* (%)	Preoperative Group I	Preoperative Group II	*P*. value	Postoperative Group I	Postoperative Group II	*P*. value
No *n* (%)	None (0%)	None (0%)	Not applicable	12 (86%)	14(100%)	0.0001***”
yes *n* (%)	14(100%)	14(100%)		2(14%)	None (0%)	

“Fisher’s exact test, p***<0.001

In terms of ocular motility limitation, the results demonstrated notable improvement in ocular motility when compared with the preoperative state. In Group I, nine patients had ocular motility limitations preoperatively, which decreased to two patients with persistent limitations at three months postoperatively. In Group II, one patient demonstrated persistent limitation of ocular motility three months after surgery, compared to seven individuals preoperatively. Regarding diplopia, three patients in Group I and six patients in Group II experienced diplopia preoperatively. Postoperatively, diplopia was completely resolved in both groups, except for one patient in Group I, who continued to experience persistent diplopia at the final follow-up (Tables 4 and 5).

**Table 4:** Ocular motility in both groups

Any limited motility, *n* (%)	Preoperative Group I	Preoperative Group I	*P*. value	Postoperative Group I	Postoperative Group II	*P*. value
No *n* (%)	5(36%)	7(50%)	0.0631ns**”**	12(86%)	13(93%)	0.1652ns**”**
yes *n* (%)	9(64%)	7(50%)		2(14%)	1(7%)	

“Fisher’s exact test, *ns* not significant

**Table 5:** Diplopia in both groups

Any diplopia, *n* (%)	Preoperative Group I	Preoperative Group II	*P*. value	Postoperative Group I	Postoperative Group II	*P*. value
No *n* (%)	11 (79%)	9 (71%)	0.2529ns**”**	13 (93%)	14 (100%)	0.0140*”
yes *n* (%)	3 (21%)	6(29%)		1 (7%)	0 (0%)	

"Fisher's exact test, *ns* not significant, p*<0.05

### Radiographic results

In Group I, the mean preoperative orbital volume of the fractured orbit was 31.53 ± 1.344 cm³, which was significantly greater than the mean volume of the intact orbit (25.81 ± 1.331 cm**³**, *p* < 0.05). After reconstruction, the mean volume of the injured orbit decreased significantly to 27.48 ± 1.324 cm**³**.

**Fig. 5:**
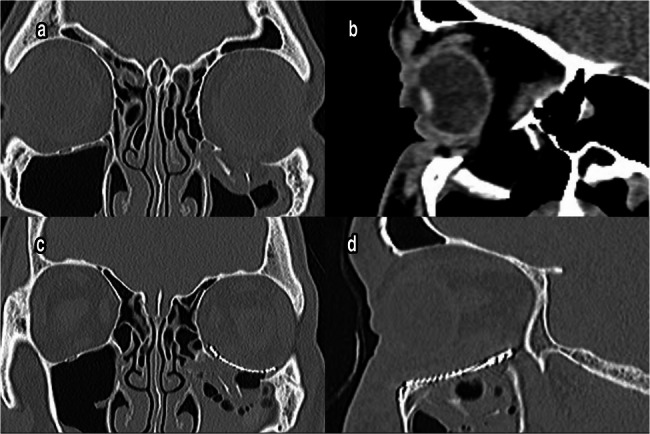
**a** & **b** Preoperative coronal and sagittal CT scan showed blow out fracture of orbital floor. **c** & **d** Reconstruction of floor using prebent titanium mesh (Group I)

**Fig. 6:**
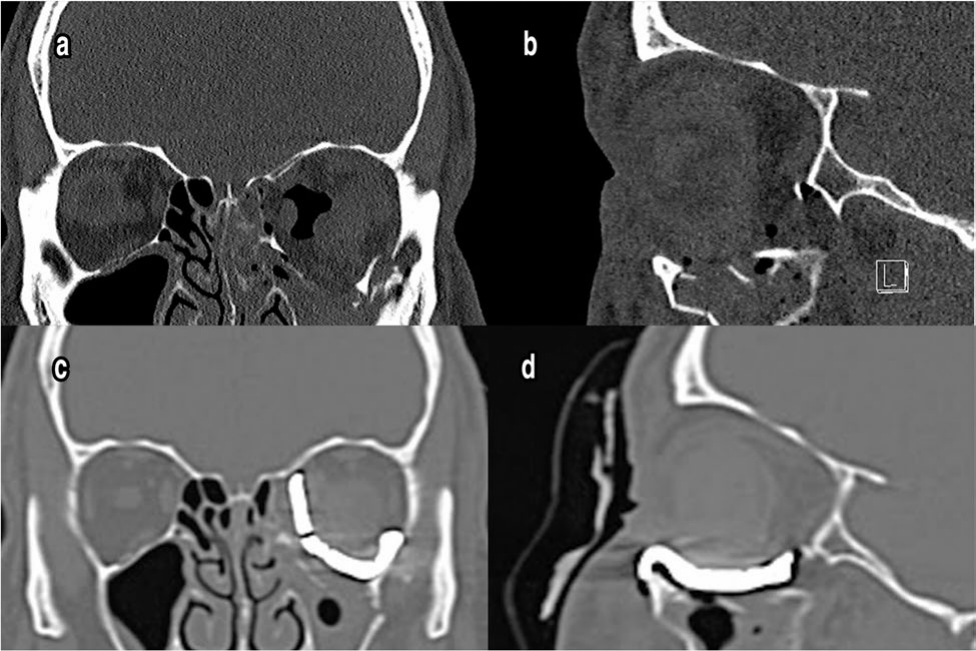
**a** & **b** Fracture and displacement of orbital walls (medial & floor) with increasing the orbital volume. **c** & **d** Postoperative CT scan showing restoration of orbital volume using titanium PSI (Group II)

In Group II, the mean volume of the damaged eye before reconstruction increased significantly to 31.41 ± 1.347 cm**³** when compared to that of the intact eye (25.05 ± 1.285 cm**³**, *p* < 0.05). After reconstruction, the mean volume of the injured eye significantly decreased to 25.62 ± 1.492 cm**³** (Figs. 5 and 6), (Table 6).

**Table 6:** Inter and Intra-Groups comparison of the mean orbital volume at different stages

		Group I	Group II	Z	*P*. value*
Preoperative volume	M ± SD Median	31.53 ± 1.344 31.87	31.41 ± 1.347 31.71	89.50	0.7131ns
Postoperative volume	M ± SD Median	27.48 ± 1.324 27.73	25.62 ± 1.492 25.71	36.00	0.0047**
Intact orbit volume	M ± SD Median	25.81 ± 1.331 26.06	25.05 ± 1.285 25.14	57.00	0.0627ns
		*P*. value•	*P*. value•		
		< 0.0001***	< 0.0001***		
		Pairwise analysis using Dunn’s test there was a statically significant between all stages (P1, P2, P3) ^€^	Pairwise analysis using Dunn’s test There was a statically significant regarding (P1, P2, P3) ^**€**^		

*Mann-Whitney test, *Z* standardized test statistic, •Friedman test

*ns* not significant, p**<0.01, p***<0.001

€ P1 difference between preoperative and postoperative volume, P2 difference between preoperative and intact, P3 difference between postoperative and intact

**Fig. 7:**
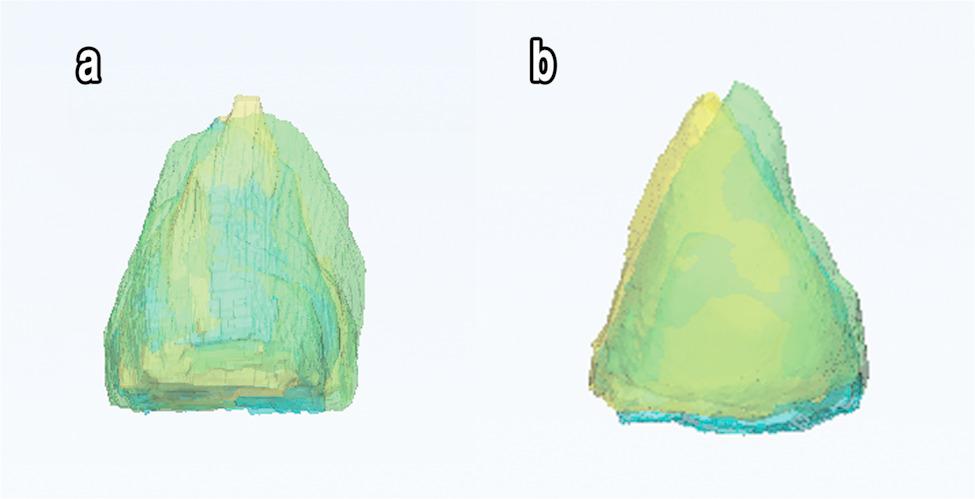
Superimposition of damaged eye (green), preoperative virtual reconstructed orbital model (yellow) over postoperative reconstructed orbit (blue), (**a**) Group I and (**b**) Group II

The mean orbital volume difference between the intact orbit and the reconstructed one was (1.666 ± 0.3884 & 0.6543 ± 0.2767 cm³) in Group I and Group II, respectively. The comparison between both groups demonstrated that the mean orbital reconstructed-to-intact volume difference was significantly higher in Group I than Group II, as *p* < 0.0001, indicating a higher precision of orbital volume restoration achieved using patient-specific implants (PSIs) (Fig. 7) and (Table 7).

**Table 7:** Comparison of the mean difference of the reconstructed-to-intact orbital volume between groups

		Group I	Group II	Z	*P*. value*
Difference	M ± SD Median	1.666 ± 0.3884 1.830	0.6543 ± 0.2767 0.6150	5.000	< 0.0001***

*Mann-Whitney test, *Z* standardized test statistic, p***<0.001

## Discussion

Fractures of the orbital floor and wall are common facial skeletal injuries [22], which may result in diplopia and enophthalmos, as well as damage to the infraorbital or optic nerve [23]. Furthermore, disruption of orbital walls can cause an increase in orbital volume, which subsequently alters globe position [24, 25]. Reconstruction of significant orbital defects is commonly recognized and widely recognized as a considerable surgical challenge due to the complicated anatomy [6]. The primary goals of orbital reconstruction are to repair orbital wall defects, restore the orbital volume, and adjust the eye position [7].

Reconstruction should be performed as atraumatically as possible to avoid further trauma to the surrounding soft tissue. Because of the extremely complex architecture, improper surgical procedures may lead to complications such as enophthalmos, diplopia, and even vision loss [26].

The most critical factor influencing both functional recovery and a satisfactory aesthetic outcome following surgery is the precise reconstruction of orbital shape with accurate volume restoration. This is especially relevant in fractures involving the orbital floor and medial walls, which form a ledge around the orbital apex and are the primary cause of inadequate treatment outcomes [14, 27, 28].

To overcome such challenges, it is crucial to adopt techniques that are safe, efficient, reproducible, and accurate. In this study, we employed computer-assisted surgery, as it represents an important advancement toward safer clinical practice and has become a routine method in recent years. As highlighted by Essig et al. [29] and Schramm et al. [30], computer-assisted technologies enable virtual surgical planning, simulation, and real-time intraoperative control.

Our study aimed to evaluate the precision of orbital wall defect reconstruction using pre-bent titanium mesh compared with customized titanium PSI. The advent of 3D printing and CAD/CAM technology allows for precise preoperative planning for orbital reconstructions. Recently, cost-effective technologies have become widely available for clinical use, allowing the fabrication of PSIs [31, 32].

Orbital reconstruction can be accomplished using a variety of implants (titanium, PTFE, silicone, and PE). In our research, we selected titanium because of its malleability, biocompatibility, and adaptability to the contour of the defect. It also promotes connective tissue integration, which stabilizes the implant and prevents migration [33].

PSIs allow for precise, digital reconstruction of orbital fractures. However, a limitation of CAD-designed implants is the manufacturing time, which can delay surgery. Zimmerer et al. [34] reported that patient-specific CAD/CAM orbital implants require 7 to 10 days to produce and are relatively expensive. In contrast, pre-bent titanium mesh, shaped using 3D patient-specific models, remains the most cost-effective method of individualized orbital wall reconstruction. The price of a 3D printer and the materials used is relatively low and affordable [35].

In our study, the mean surgical interval was 9 to 10 days for both groups. The customized orbital implants (PSIs) were delivered within 5–7 days, ensuring that the surgery was not delayed. Similarly, Dal Canto et al. [36] reported no significant differences in the occurrence of enophthalmos or diplopia between early (within two weeks) and delayed surgical correction.

It is worth emphasizing that the fabrication of PSIs mainly requires engineering time, rather than surgical or operating room time [7, 34, 35, 37]. From our experience, both PSIs and prebent titanium plates were technically straightforward to use and required similar operative times.

In the present study, we demonstrated that using CAD-CAM PSIs significantly improved vertical globe position and enophthalmos compared with the prebent plates. These findings are consistent with the outcomes reported by Zimmerer et al. [34] and Chepurnyi et al. [38], while no statistically significant differences were observed in visual acuity or ocular motility between the two groups.

Although dimensionally stable titanium PSIs demonstrated greater reconstruction accuracy, this did not translate into better clinical outcomes in all cases of our study. Zimmerer et al. [34] explained that, in addition to precision of reconstruction, a wide range of other factors influence the postoperative orbital function, such as fat atrophy and soft tissue changes.

Our study found a significant decrease in orbital volume in both groups following surgery. The mean orbital volume difference between reconstructed and intact orbits after reconstruction with a pre-bent titanium plate was 1.666 ± 0.3884 cm³, which is consistent with previous reports documenting differences ranging from 1.6 to 2.7 cm³ [34, 37, 38]. In contrast, PSI reconstruction resulted in a smaller mean volume difference of 0.6543 ± 0.2767 cm³. Chepurnyi et al. [38] reported similar results, noting a mean difference of 0.74 ± 0.6 cm³ after PSI surgery.

These results demonstrate that PSIs are more effective than prebent plates in restoring the volume of extensive orbital defects. Although they are more cost, the structural stability of PSIs enables for precise reconstruction of delicate orbital regions, including the posterior ledge and the transition zone. These structures are considered vital for successful reconstruction [39]. Consistent with the results of Chen et al. [40] and Cole et al. [41], postoperative 3D imaging demonstrated symmetrical orbital reconstructions when PSIs were used with meticulous reconstruction of the transition zone and the posterior ledge.

In addition, virtual surgical planning enables the assessment of implant placement in relation to the orbital walls, nerves, and ocular muscles. Design modifications can be made preoperatively if conflicts are detected, thereby reducing intraoperative complications and the need for revision surgery [35, 42].

Our results indicate that PSIs provide high accuracy, favorable clinical outcomes in large orbital defects, and reduce the likelihood of postoperative revision. The use of CAD-CAM technology in PSI manufacturing enables surgeons to finalize reconstruction plans before entering the operating room, and the stiffness of PSIs prevents implant deformation during placement. Gander et al. [16] and Kärkkäinen et al. [43] likewise demonstrated that PSIs restore orbital volume and correct residual enophthalmos effectively.

Although pre-bent titanium plates are more cost-effective and readily available, they are often inadequate for large or complex orbital defects, where precise anatomical restoration is required. Schlittler et al. [11] reported a significantly higher revision rate in such cases due to implant malposition. This may explain the lower incidence of residual enophthalmos in the PSI group in our study. These results align with findings from multiple studies that reported better outcomes and lower revision rates with PSIs compared to standard or manually bent implants [11, 16, 34]. However, the Consorti et al. [44] found that 3D preformed titanium meshes achieved reconstruction of inferomedial fractures with accuracy comparable to customized implants, with both groups demonstrating similar clinical outcomes. This may be attributed to the use of intraoperative navigation, which likely provided an additional advantage for the preformed mesh group.

Our results highlight the clinical value of using PSIs in large orbital fractures involving multiple walls. Their precise design and stability allow for effective orbital volume restoration. In contrast, malleable pre-bent plates may be more suitable for single wall fractures, where the risk of malposition is lower. This is consistent with findings of Schlittler et al. [45] and Schreurs et al. [46], who concluded that PSIs are better suited for larger and more complex defects. Similarly, Chepurnyi et al. [10] reported that while prebent implants are comparable to PSIs in cases with preserved infraorbital support and retrobulbar structure, they are less effective in extensive reconstructions.

### Study Limitations

The primary limitation of this study was the relatively small sample size, although this did not seem to affect the comparative outcomes between the two groups. Additionally, the short follow-up period limited the assessment of long-term functional and aesthetic outcomes, such as persistent diplopia and late-onset enophthalmos. Furthermore, all surgeries were performed by two oral and maxillofacial surgeons, which may have introduced possible operator bias, limiting the external validity of the results. Future studies with larger cohorts and longer follow-up durations are warranted to validate these findings.

## Conclusion

CAD/CAM patient-specific implants (PSIs) demonstrated higher precision and favorable clinical efficacy, especially in the reconstruction of large orbital fractures. They reduced the need for surgical revision and should be considered a more accurate alternative to prebent plates in complex cases. Conversely, prebent plates may represent a more cost-effective option for single-wall fractures, where the risk of implant malposition is lower.

## Supplementary Information

Supplementary Material 1.

Supplementary Material 2.

## Data Availability

Upon reasonable request, the datasets used and/or analyzed in the present study are available from the corresponding author.

## Abbreviations

DICOM: Digital Imaging and Communications in Medicine
PSIs: Patient-specific implants
CAD/CAM: Computer-aided design and computer-aided manufacturing
CT: Computed Tomography
STL: Stereolithography
CNC: Computer numerical control
