# Supplementary material for: The accuracy of reconstruction of orbital wall fractures using prebent mesh versus patient specific implants: a randomized clinical trial
Source: BMC Oral Health. 2025 Nov 1;25:1719. doi: 10.1186/s12903-025-07082-z (PMC12579402; doi:10.1186/s12903-025-07082-z)

**Image Processing and Segmentation**

- DICOM files were imported into [**Mimics Innovation Suite v21** (Materialise, Leuven, Belgium)
- Bone segmentation of the orbital cavity was performed using a consistent Hounsfield Unit (HU) threshold (typically >226 HU).
- Manual refinement was applied to delineate the bony boundaries of the orbit plus segmentation of orbital volume as a separate entity.
- For each case, both preoperative and postoperative scans were segmented following the same protocol to ensure comparability.

**Generation of 3D Models**

- Segmented orbital cavities were converted into 3D surface meshes and exported in STL format. To ensure geometric consistency, minor surface irregularities were smoothed using built-in software tools with smoothing settings kept constant across all models.
- This was done for pre - planned - post operative orbital volumes

Pre op


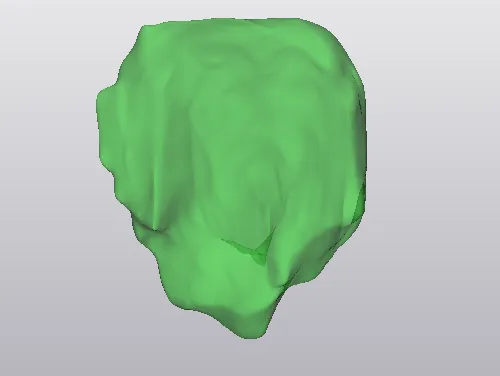


Planned


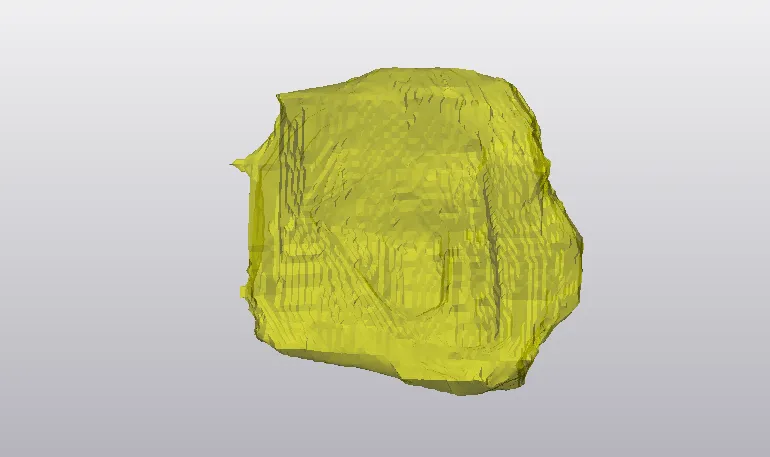


Post op


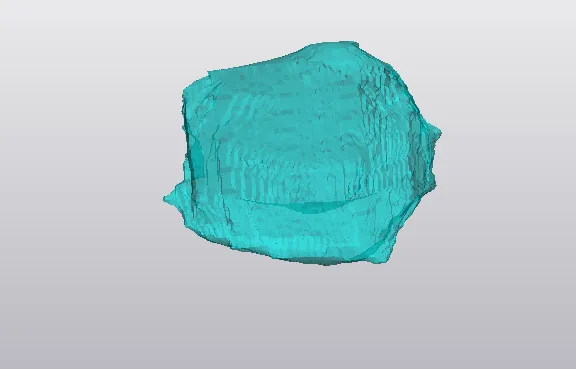


**Model Alignment and Registration**

To facilitate volumetric comparison, all models were registered to a common cranial reference frame using rigid registration based on anatomical landmarks, including the nasion, frontozygomatic suture, and anterior nasal spine. This ensured spatial alignment of the preoperative and postoperative datasets within the same coordinate system.

In cases of unilateral fractures, the intact contralateral orbit was mirrored across the midsagittal plane to serve as an anatomical reference for restoration.

**Orbital Volume Measurement**

The volumes of the preoperative, postoperative, and mirrored orbits were computed using the volumetric analysis module within [Mimics or relevant software]. The computed volumes were expressed in cubic millimeters (mm³). To ensure accuracy, all volume measurements were performed by a single operator and repeated twice; the average was recorded for analysis.


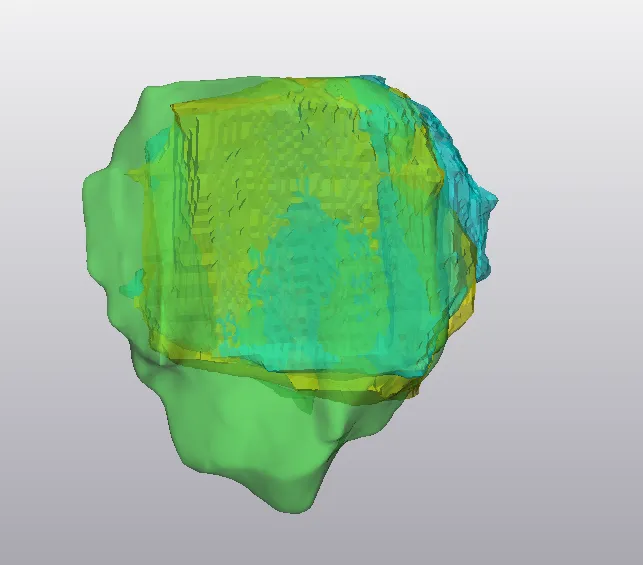

Supplement: Supplementary file 2 — Supplementary Material 2. [file 12903_2025_7082_MOESM2_ESM.docx]
